# Effector T Helper Cells Are Selectively Controlled During Pregnancy and Related to a Postpartum Relapse in Multiple Sclerosis

**DOI:** 10.3389/fimmu.2021.642038

**Published:** 2021-03-15

**Authors:** Steven C. Koetzier, Rinze F. Neuteboom, Annet F. Wierenga-Wolf, Marie-José Melief, C. Louk de Mol, Angelique van Rijswijk, Willem A. Dik, Bieke Broux, Ronald van der Wal, Sjoerd A. A. van den Berg, Joost Smolders, Marvin M. van Luijn

**Affiliations:** ^1^Department of Immunology, Erasmus MC, University Medical Center Rotterdam, Rotterdam, Netherlands; ^2^MS Center ErasMS, Erasmus MC, University Medical Center Rotterdam, Rotterdam, Netherlands; ^3^Department of Neurology, Erasmus MC, University Medical Center Rotterdam, Rotterdam, Netherlands; ^4^Laboratory Medical Immunology, Department of Immunology, Erasmus MC, University Medical Center Rotterdam, Rotterdam, Netherlands; ^5^Neuro-Immune Connections and Repair Lab, Department of Immunology and Infection, Biomedical Research Institute, University MS Center, Hasselt University, Hasselt, Belgium; ^6^Department of Clinical Chemistry, Erasmus MC, University Medical Center Rotterdam, Rotterdam, Netherlands; ^7^Department of Internal Medicine, Erasmus MC, University Medical Center Rotterdam, Rotterdam, Netherlands; ^8^Neuroimmunology Researchgroup, Netherlands Institute for Neuroscience, Amsterdam, Netherlands

**Keywords:** relapse risk, third trimester, serum-related factors, hormones, inflammatory cytokine potential

## Abstract

**Background:** Multiple sclerosis (MS) patients are protected from relapses during pregnancy and have an increased relapse risk after delivery. It is unknown how pregnancy controls disease-contributing CD4^+^ T helper (Th) cells and whether this differs in MS patients who experience a postpartum relapse. Here, we studied the effector phenotype of Th cells in relation to pregnancy and postpartum relapse occurrence in MS.

**Methods:** Memory skewing and activation of effector Th subsets were analyzed in paired third trimester and postpartum blood of 19 MS patients with and without a postpartum relapse and 12 healthy controls. *Ex vivo* results were associated with circulating levels of pregnancy-induced hormones and mirrored *in vitro* by exposing proliferating Th cells to corresponding serum samples.

**Results:** Based on HSNE-guided analyses, we found that effector memory proportions of Th cells were increased in postpartum vs. third trimester samples from MS patients without a postpartum relapse. This was not seen for relapsing patients or healthy controls. CXCR3 was upregulated on postpartum memory Th cells, except for relapsing patients. These changes were verified by adding sera from the same individuals to proliferating Th cells, but did not associate with third trimester cortisol, estradiol or progesterone levels. For relapsing patients, activated memory Th cells of both third trimester and postpartum samples produced higher levels of pro-inflammatory cytokines.

**Conclusion:** Effector Th cells are differentially regulated during pregnancy in MS patients, likely via serum-related factors beyond the studied hormones. The pro-inflammatory state of memory Th cells during pregnancy may predict a postpartum relapse.

## Introduction

Women with multiple sclerosis (MS) have a reduced relapse rate of ~70% in the third trimester of pregnancy (1), which coincides with high levels of pregnancy-induced hormones, such as estradiol, progesterone and cortisol (2). Vice versa, MS patients have an increased relapse risk 4–8 weeks after delivery (postpartum) (1), a period in which such hormones rapidly decline (3). Since an increased relapse rate is associated with worsening of disease (4), in-depth insights into pregnancy as a naturally occurring disease modifier will help to better understand and predict the heterogeneous disease course of MS.

In the earliest phase of MS, CD4^+^ T helper (Th) and not CD8^+^ T cell clonotypes are reduced in the blood (5) and enriched in the cerebrospinal fluid (CSF) (6). This suggests that Th cells are the first to enter the central nervous system (CNS) of MS patients and trigger disease activity. Similar trends have been observed during pregnancy (7), indicating that alterations in Th cell composition may predict relapse occurrence in the early postpartum period. A notable downside of clonotype tracking is the diverse nature of Th cell clones between individuals, making it impossible to identify universal repertoires for predicting MS disease activity. In experimental autoimmune encephalitis, the addition of pregnancy-related hormones dampens disease activity (8–10), whereas reduced basal levels aggravate the disease course (11, 12). Although these observations are linked to altered effector Th cell functions (8–12), the exact impact of pregnancy on *ex vivo* effector Th cells in MS patients and how this corresponds to increased postpartum relapse risk remains to be elucidated.

In this study, we compared memory skewing and activation of pro-inflammatory Th cells between paired third trimester and early postpartum blood from MS patients with and without a postpartum relapse as well as healthy controls. Fluctuations in effector Th phenotype were associated with pregnancy-related serum factors both *ex vivo* and *in vitro*.

## Materials and Methods

### Blood Sampling

Blood was collected using Vacutainer SST for serum, and CPT tubes (BD Biosciences, Erembodegem, Belgium) containing sodium heparin for cell-based analysis. Serum and peripheral blood mononuclear cells (PBMCs) were isolated according to manufacturer's instructions. PBMCs were taken up in RPMI 1640 (Lonza, Verviers, Belgium) containing 20% fetal calf serum (Thermo Fisher Scientific, Landsmeer, The Netherlands) and 10% dimethyl sulfoxide (Sigma-Aldrich, St Louis, MO, USA) and stored in liquid nitrogen until further use. Serum samples were stored at −80°C.

### Antibodies, Flow Cytometry, and Analysis

The fluorescently labeled anti-human monoclonal antibodies used for flow cytometry are shown in Supplementary Table 1. In all experiments, viable cells were analyzed using Fixable Viability Stain 700 (BD Biosciences) or Fixable Viability Dye eFluor 520 (Thermo Fisher Scientific) by performing staining for 15 min at 4°C in the dark. Surface markers were stained for 30 min. Cells were measured using the LSRII-Fortessa (BD Biosciences) and analyzed using both Cytosplore and BD FACSDiva (version 8.0.1) software. For Hierarchical Stochastic Neighbor Embedding (HSNE) analysis in Cytosplore (13), events were downscaled to a maximum of 80,000 per sample. PBMCs of non-relapsing MS patients and healthy controls were measured in the same experiments. PBMCs of relapsing MS patients were measured a year later and thus excluded from HSNE analysis due to the effect of fluorescent intensity shifts on this type of analysis. We excluded Tregs (CD25^high^CD127^low^) and defined naive, central memory and effector memory populations based on CCR7 and CD45RA expression. Th1, Th2, Th17, Th17.1 and Th17 “double-positive” (DP) subsets were discriminated based on differential expression of CCR6, CCR4, and CXCR3 (14, 15).

### Serum-Based Th Cell Proliferation Assays

CD4^+^ cells were isolated from the blood of healthy non-pregnant females (Sanquin, Amsterdam, The Netherlands) using CD4 microbeads and the autoMACS Pro Separator (both Miltenyi Biotec, Bergisch Gladbach, Germany), frozen as described above and stored in liquid nitrogen until further use. Thawed cells were labeled with CFSE (1:20,000; CFDA-SE, Molecular Probes via Thermo Fisher Scientific) for 10 min at 37°C. After washing twice with RPMI 1640 containing 100 U/ml penicillin and 100 μg/ml Streptomycin (now termed as “Pen/Strep”; Lonza) and 5% FCS (Thermo Fisher Scientific), cells were plated at a concentration of 1 × 10E^6^/ml in RPMI1640 containing Pen/Strep. Anti-CD3/-CD28 dynabeads (Thermo Fisher Scientific) were added (1:5) together with paired third trimester or postpartum serum samples from MS patients or healthy controls till a concentration of 5%. After 72 h of culture, dynabeads were removed and cells were assessed by flow cytometry.

### Cytokine Measurement

Memory Th cells (CD3^+^CD4^+^CD8^−^CD25^−/*dim*^CD45RA^−^) were isolated using a FACSAria-III machine (BD Biosciences) and plated at a concentration of 0.5 × 10^6^/ml in RPMI 1640 containing 5% inactivated human AB serum (Sanquin) and Pen/Strep. Cells were rested overnight (37°C) and were stimulated with phorbol 12-myristate 13-acetate (PMA; 1:2,000) and ionomycin (1:500; both Sigma-Aldrich) for 5 h. Subsequently, supernatants were harvested and stored at −80°C until further use. Supernatants were diluted 2-fold and analyzed for GM-CSF, IFN-γ, IL-2, IL-6, IL-17A and TNF-α using a custom Luminex multiplex bead immunoassay (R&D Systems, Abingdon, UK). Measurements were performed on a Bio-Plex MAGPIX machine and data were analyzed using Bio-Plex Manager MP software (both Bio-Rad, Hercules, California, USA).

### Hormone Measurements

Both cortisol and progesterone levels were measured by ultraperformance liquid chromatography-tandem mass spectrometry (UPLC-MS/MS; Waters TQS, Waters, Etten-Leur, The Netherlands). Both steroids were calibrated using commercially available calibrators (Cerilliant Corporation, Round Rock, TX, USA); article number P-069 (progesterone) and C-106 (cortisol). Trueness was verified for progesterone using reference materials ERM-DA347 and ERM-DA348. For cortisol, trueness was verified using the ERM-DA451 IFCC cortisol reference serum panel (34 levels). Both analytes were measured without bias when compared to the reference materials. Measurement uncertainty was assessed for both analytes by measurement of tri-level matrix matched controls (UTAK, Valencia, CA, USA). For progesterone, the coefficients of variation (CVs) over a period of 1 year were 5.2, 6.6, and 10.4% at 1.4, 17.9, and 65.3 nM, respectively. For cortisol, CVs were 3.6, 3.7, and 4.0% at 40.0, 98.6, and 573.0 nM, respectively. Estradiol levels were measured by automated immunoassay on (Fujirebio Lumipulse G1200, Fujirebio, Tokyo, Japan). CVs over 19 runs were 4.4 and 3.9% at 345 and 1,099 pM, respectively.

### Statistics

Generalized linear mixed models (GLMM) with a gamma distribution were used to assess longitudinal effects and the false discovery rate (FDR) method of Benjamini and Hochberg (BH) was used to correct for multiple testing (R statistical software package version 4.0). Data between clinical groups were compared using Kruskal-Wallis tests incorporating Dunn's multiple comparisons or 2-way analysis of variance (ANOVA) with Bonferroni's multiple comparison tests (GraphPad Prism software version 8, San Diego, CA, USA). Longitudinal results were displayed as connected data points for each individual. For the comparison of data between clinical groups, Box and Whisker plots were used containing individual data points and displaying the median with the interquartile range (IQR). For all tests, a *p*-value of < 0.05 (^*^) after multiple testing correction was considered significant. Categorical data were analyzed using Chi-squared, Kruskal-Wallis or Wilcoxon rank-sum tests.

## Results

### Study Samples

In this exploratory study, pregnant relapsing-remitting MS patients visiting MS center ErasMS were included and retrospectively validated to match the most recent McDonald 2017 criteria (16, 17). Exclusion criteria were history of recurrent abortion, hypertension and diabetes mellitus. Patients did not receive disease modifying treatment at least 3 months prior to pregnancy and during this study. Third trimester expanded disability status scale (EDSS) scores were similar between patients that did or did not experience a clinically-defined postpartum relapse, as described previously (17) (Table 1). Patients did not experience relapses during pregnancy. Postpartum EDSS scores did differ significantly between non-relapsing and relapsing MS patients (Table 1). Unfortunately, radiological data were not available. Healthy pregnant woman were recruited from the outpatient obstetric clinic at the Erasmus MC. Patient groups and healthy controls were matched for age and selected based on the availability of frozen PBMCs at 28–30 weeks of pregnancy (third trimester) and 4–8 weeks after delivery (postpartum). Other clinical characteristics are summarized in Table 1. The only difference between the healthy controls and the MS groups were the amount of females giving birth for their first time (nullipara).

**Table 1:** Clinical information of pregnant MS patients and healthy controls.

	***Ex vivo*** **(Th cells)**			
	MS-NR^*,**^	MS-R^*,**,***^	**HC**	***P*-value**
Number of individuals	13.0	6.0	12.0	NA
Median maternal age with IQR	33.3 (26.8–33.8)	32.1 (29.8–33.7)	33.3 (27.7–34.1)	0.94
Median EDSS third trimester with IQR	1.5 (1.0–1.5)	1.0 (0.3–1.8)	NA	0.82
Median EDSS postpartum with IQR	1.0 (0.0–1.5)	1.8 (1.5-2.8)	NA	0.02
Nullipara	4	1	9	0.03
Cesarean section	2	0	1	0.60
(Pre)eclampsia	0	0	0	NA
Median gestation (weeks) with IQR	40.0 (39.0–41.0)	38.0 (38.0–39.0)	39.0 (37.0–40.0)	0.13
	***In vitro*** **(sera)**			
	MS-NR^*,**^	MS-R^*,**,***^	**HC**	***P*****-value**
Number of individuals	8.0	5.0	8.0	NA
Median maternal age with IQR	33.5 (26.6–34.6)	31.5 (29.2–34.1)	33.3 (31.0–34.1)	0.91
Median EDSS third trimester with IQR	1.3 (0.8–1.6)	1.0 (0.0–1.6)	NA	0.94
Median EDSS postpartum with IQR	1.3 (0.8–1.6)	2.0 (1.5–3.0)	NA	0.12
Nullipara	3	0	6	0.04
Cesarean section	1	0	1	0.76
(Pre)eclampsia	0	0	0	NA
Median gestation (weeks) with IQR	40.0 (38.8–41.3)	38.5 (38.0–39.3)	38.5 (36.3–40.0)	0.29
	**Luminex** (memory Th cells)			
	MS-NR^*,**^	MS-R^*,**,***^	**HC**	***P*****-value**
Number of individuals	6.0	6.0	7.0	NA
Median maternal age with IQR	35.5 (33.4–37.3)	32.1 (29.8–33.7)	34.0 (32.9–35.5)	0.30
Median EDSS third trimester with IQR	1.3 (0.3–1.5)	1.0 (0.3–1.8)	NA	1.00
Median EDSS postpartum with IQR	1.3 (0.3–1.5)	1.8 (1.5–2.8)	NA	0.12
Nullipara	2	1	5	0.17
Cesarean section	1	0	1	0.64
(Pre)eclampsia	0	0	0	NA
Median gestation (weeks) with IQR	40.5 (38.5–41.8)	38.0 (38.0–39.0)	38.0 (37.0–39.5)	0.13
	**UPLC-MS/MS (sera)**			
	MS-NR^*,**^	MS-R^*,**,***^	**HC**	***P*****-value**
Number of individuals	13.0	6.0	12.0	NA
Median maternal age with IQR	33.3 (26.8–33.8)	32.8 (29.8–35.4)	33.3 (27.7–34.1)	0.99
Median EDSS third trimester with IQR	1.5 (1.0–1.5)	1.5 (0.3–2.0)	NA	0.71
Median EDSS postpartum with IQR	1.0 (0.0–1.5)	2.0 (1.6–2.8)	NA	0.02
Nullipara	4	1	9	0.04
Cesarean section	2	0	1	0.60
(Pre)eclampsia	0	0	0	NA
Median gestation (weeks) with IQR	40.0 (39.0–41.0)	39.0 (38.0–39.0)	39.0 (37.0–40.0)	0.18

* *Did not experience clinically-defined relapses during pregnancy*.

** *Patients did not receive immune modifying treatment for at least 3 months prior to pregnancy and until the end of this study*.

*** *Information regarding nullipara, caesaraean section, (pre) eclampsia and gestation is missing for one RRMS patient*.

*MS-NR, RRMS patients without clinically-defined relapses in the early postpartum period (4–8 weeks); MS-R, RRMS patients with clinically-defined relapses in the early postpartum period (4–8 weeks); EDSS, expanded disability status scale; IQR, interquartile range*.

### The Influence of Pregnancy and Pregnancy-Associated Serum Factors on Th Memory Cell Phenotype in MS

**Figure 1:**
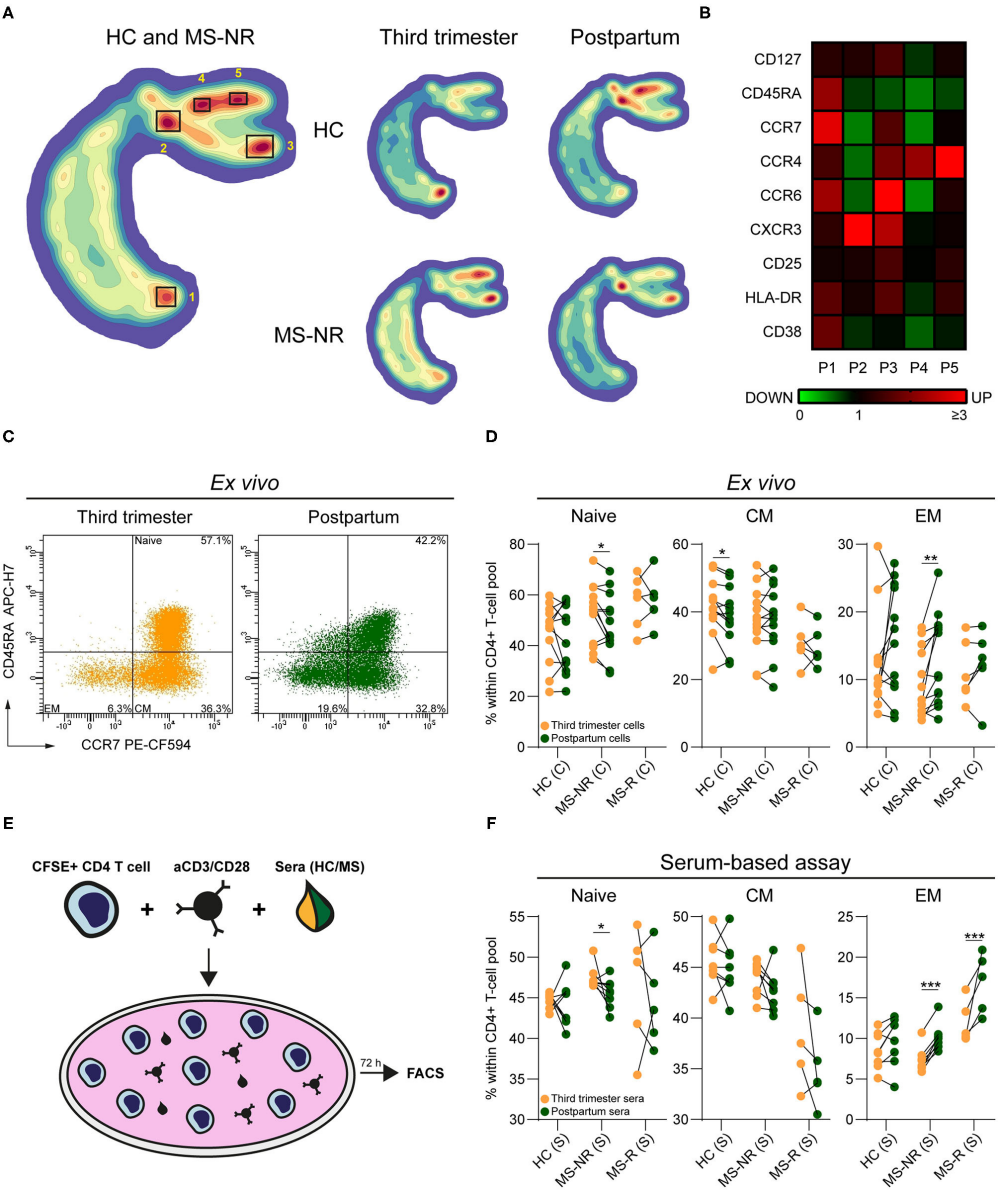
The impact of pregnancy on memory Th cells from distinct clinical groups. **(A)** HSNE density plot of paired HC (*n* = 12) and MS-NR (*n* = 13) third trimester and postpartum Th cells (CD25^−/*dim*^CD127^+^). **(B)** Expression heatmap of the markers used for the HSNE analysis within each Th cluster. The changes in expression (green = downregulated, black = no change, red = upregulated) are based on the median fluorescence intensity (MFI) per marker, which are normalized to the MFI's of the markers within the entire Th pool. **(C)** Representative FACS plot showing the distribution of naive, central memory (CM), effector memory (EM) subsets within Th cells from paired third trimester and postpartum blood of a MS-NR patient. **(D)** Naive, CM and EM frequencies within Th cells of paired third trimester and postpartum blood of (HC, *n* = 12; MS-NR, *n* = 13 and MS-R, *n* = 6). **(E)** Graphical illustration of the serum-based assay. Healthy female CD4^+^ T cells were labeled with CFSE and activated with aCD3/CD28 beads for 72 h in the presence of third trimester or postpartum sera from each clinical group. **(F)** Frequencies of naive, CM and EM Th subsets after exposing activated CD4^+^ T cells to paired third trimester and postpartum sera for 72 h (HC, *n* = 8; MS-NR, *n* = 8 and MS-R, *n* = 5). Data were compared using GLMM with FDR-BH correction. ^*^p < 0.05, ^**^p < 0.01, and ^***^p < 0.001. “HC” = healthy controls, “MS-NR” = MS patients without a postpartum relapse, “MS-R” = MS patients with a postpartum relapse, “C” = cells and “S” = sera.

To assess shifts in phenotypic effector profiles, we first analyzed *ex vivo* flow cytometry data of CD4^+^ Th cells from paired third trimester and early postpartum blood of healthy controls (*n* = 12; HC) and MS patients without a postpartum relapse (*n* = 13; MS-NR) in an unbiased manner using HSNE (Figure 1A and Supplementary Figure 1). For both the HC and MS-NR group, cluster P2 (CCR7^low^CD45RA^low^) was decreased, while cluster P1 was increased (CCR7^high^CD45RA^high^) in third trimester vs. postpartum blood (Figures 1A,B). Cluster P3 (CCR7^dim^CD45RA^low^) was proportionally decreased during pregnancy relative to the presence of other clusters. For the MS-NR group, this was confirmed by manual gating, showing significantly reduced effector memory (CCR7^low^CD45RA^low^) and increased naive (Figures 1C,D; *p* = 0.002 and *p* = 0.012) Th cell frequencies. For the HC group, these frequencies did not differ (Figure 1D) and were unrelated to nullipara count (Table 1 and data not shown). In 6 MS patients who did experience a postpartum relapse (MS-R), no significant differences were found in memory Th cell phenotype between third trimester and postpartum samples (Figure 1D). To determine whether pregnancy-relevant factors present in the blood (e.g., hormones) contributed to these phenotypes, we added corresponding serum samples to activated healthy female CD4^+^ Th cells *in vitro* (Figure 1E). Th cells exposed to paired MS-NR and HC sera showed similar changes in phenotype skewing as found *ex vivo* (Figure 1F; *p* = 0.026 and *p* < 0.001). In contrast to our *ex vivo* results (Figure 1D), reduced frequencies of effector memory Th cells were also seen after exposure to third trimester compared to postpartum MS-R sera (Figure 1F; *p* < 0.001). Interestingly, these frequencies appeared higher than in the MS-NR and HC group. Overall, these data suggest that effector memory Th cells are kept in check during pregnancy by serum-related factors, which is lost and may contribute to an MS relapse early after delivery.

### Pregnancy-Related Effects on CXCR3 and CCR6 Expression by Memory Th Cells in MS

**Figure 2:**
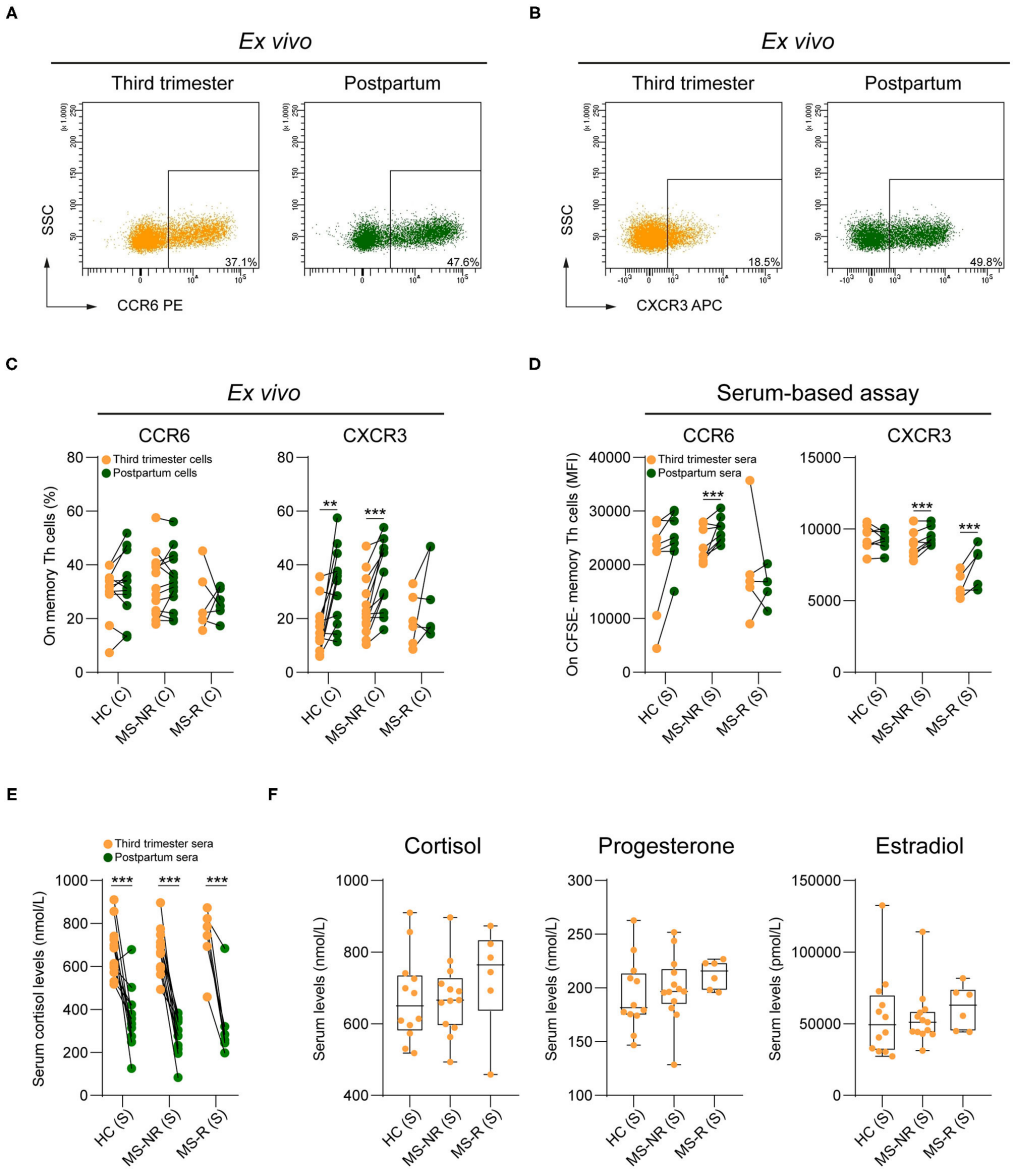
The impact of pregnancy on CXCR3 and CCR6 expression by memory Th cells from different clinical groups. Representative FACS plot displaying CCR6 **(A)** and CXCR3 **(B)** expression on memory Th cells in paired third trimester and postpartum blood of a MS-NR patient. **(C)** Frequencies of CCR6^+^ and CXCR3^+^ counterparts within the pool of memory Th cells in paired third trimester and postpartum blood (HC, *n* = 12; MS-NR, *n* = 13 and MS-R, *n* = 6). **(D)** CCR6 and CXCR3 expression (median fluorescent intensity; MFI) on activated healthy donor CFSE^−^ Th cells exposed to paired third trimester and postpartum sera for 72 h (HC, *n* = 8; MS-NR, *n* = 8 and MS-R, *n* = 5). **(E)** Cortisol levels (nmol/L) in paired third trimester and postpartum sera and **(F)** third trimester cortisol, progesterone (nmol/L) and estradiol (pmol/L) levels (HC, *n* = 12; MS-NR, *n* = 13 and MS-R, *n* = 6). Data were compared using GLMM with FDR-BH correction and Kruskal-Wallis with Dunn's multiple comparison tests. **p < 0.01 and ***p < 0.001. “HC” = healthy controls, “MS-NR” = MS patients without a postpartum relapse, “MS-R” = MS patients with a postpartum relapse, “C” = cells and “S” = sera.

According to the HSNE analysis, effector memory (CCR7^dim/low^CD45RA^low^) Th clusters P2 and P3 (Figure 1B) mainly expressed CXCR3, a hallmark of IFN-γ-producing subsets (18). Cluster P3 also showed upregulation of Th17 lineage marker CCR6 (14). Manual gating revealed that CXCR3 and not CCR6 expression was lower in third trimester compared to postpartum samples (Figures 2A–C). This was significant for both HC (*p* = 0.002) and MS-NR groups (*p* < 0.001), but not for the MS-R group. In line with our HSNE analysis (Figure 1B), these differences were seen for all CXCR3-expressing Th subsets including Th1 (CCR6^−^CXCR3^+^CCR4^−^), Th17 DP (CCR6^+^CXCR3^+^CCR4^+^) and Th17.1 (CCR6^+^CXCR3^+^CCR4^−/*dim*^) cells (Supplementary Figure 2). Interestingly, only MS-associated Th17.1 cells (19) were decreased in MS-R postpartum blood and showed a prominent effector memory phenotype in comparison to other Th subsets (see Supplementary Figures 2, 3). In sharp contrast to the HC group, CXCR3 was downregulated on *in vitro* proliferating memory Th cells exposed to MS-NR and MS-R third trimester vs. postpartum sera (Figure 2D; *p* < 0.001). This was comparable to the *in vitro* effects on effector memory phenotypes (Figure 1F). Although no differences were seen on *ex vivo* Th memory cells from patients and controls, CCR6 expression levels were reduced *in vitro* after the addition of third trimester compared to postpartum MS-NR sera (Figure 2D; *p* < 0.001). As expected, mass spectrometry revealed that cortisol levels were strongly elevated in third trimester sera from each clinical group (Figure 2E; *p* < 0.001). Progesterone and estradiol could not be detected in postpartum samples. Third trimester cortisol, progesterone and estradiol levels did not differ between clinical groups (Figure 2F). These data indicate that CXCR3^+^ memory Th cells are selectively controlled by serum-related factors in pregnant MS patients, which cannot be explained by changes in cortisol, progesterone or estradiol serum levels between clinical groups.

### The Association of the Pro-Inflammatory State of Memory Th Cells With Pregnancy in MS

**Figure 3:**
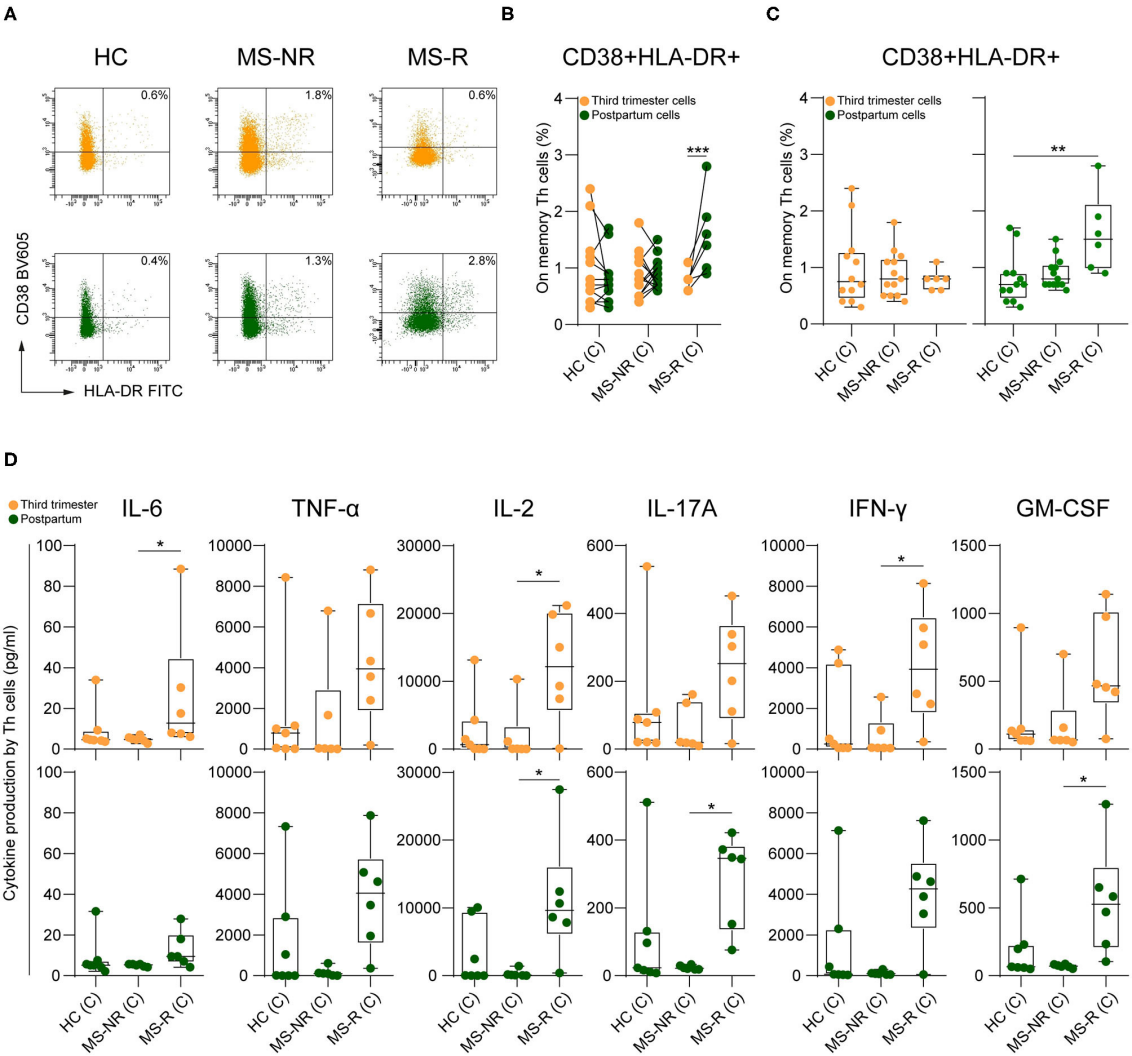
The impact of pregnancy on the activation of pro-inflammatory memory Th cells. **(A)** Representative FACS plot displaying CD38 and HLA-DR co-expression on memory Th cells in paired third trimester and postpartum blood. (**B,C**) CD38^+^HLA-DR^+^ frequencies within the memory Th pool between paired third trimester and postpartum blood per group and per period between groups (HC, *n* = 12; MS-NR and *n* = 13 and MS-R, *n* = 6). **(D)** IL-6, TNF-α, IL-2, IL-17A, IFN-γ, and GM-CSF production (pg/ml) by PMA/ionomycin-stimulated memory Th cells of third trimester and postpartum blood. Cytokines were measured in the culture supernatants and determined by Luminex (HC, *n* = 7; MS-NR, *n* = 6, and MS-R, *n* = 6). Data were compared using GLMM with FDR-BH correction and Kruskal-Wallis with Dunn's multiple comparison tests. *p < 0.05, **p < 0.01, and ***p < 0.001. “HC” = healthy controls, “MS-NR” = MS patients without a postpartum relapse, “MS-R” = MS patients with a postpartum relapse, “C” = cells.

Finally, we explored whether the activation and pro-inflammatory capacity of memory Th cells is differentially regulated in our clinical groups. For this purpose, co-expression of CD38 and HLA-DR was investigated on third trimester and postpartum memory Th cells (Figure 3A). Only in the MS-R group, the proportions of CD38^high^HLA-DR^high^ memory Th cells were increased in postpartum compared to third trimester samples (Figure 3B; *p* < 0.001). These proportions were higher than those in the HC group (Figure 3C; *p* = 0.008). To address how this is related to their ability to produce pro-inflammatory cytokines, memory Th cells were purified from paired third trimester and postpartum samples and stimulated with PMA and ionomycin. Although not significant for each cytokine, higher levels of interleukin-6 (IL-6), tumor necrosis factor α (TNF-α), IL-2, IL-17A, interferon gamma (IFN-γ) and granulocyte-macrophage colony-stimulating factor (GM-CSF) were found in supernatants from postpartum memory Th cells of the MS-R group (Figure 3D). Notably, Th1-associated cytokine IFN-γ and not Th17-associated cytokine IL-17A pre-dominated these samples. Importantly, the same was true for cells derived from third trimester blood in this clinical subgroup (Figure 3D). No significant differences in cytokine production were seen between paired third trimester and postpartum samples from each clinical subgroup (see Supplementary Figure 4). These findings reveal that pro-inflammatory memory Th cells of MS patients with a future postpartum relapse are more trigger-happy already during the third trimester of pregnancy.

## Discussion

There is substantial evidence that pathogenic effector Th cells are suppressed during pregnancy in MS patients (7, 20, 21). However, it remains elusive which factors drive this phenomenon and currently no biomarkers are available that accurately predict which patients will experience a relapse after pregnancy. For this purpose, we assessed the effector phenotype of disease-relevant Th subsets in relation to hormones and postpartum relapse occurrence in MS. Our work reveals that effector memory Th subsets are suppressed during pregnancy and rise in frequencies early after delivery, which is controlled by unidentified serum factors in MS. Their increased *in vitro* pro-inflammatory capacity during pregnancy and reduced *ex vivo* frequencies puts CXCR3^+^ memory Th cells forward as a potential marker for predicting a postpartum relapse in MS.

Woman are more prone to develop Th1 responses (22). This may not only contribute to the increased prevalence of autoimmune diseases such as MS in females, but also provide a reason why the immune system shifts from a Th1- to a Th2-like state during pregnancy (23). Consistently, we found that the frequencies of *ex vivo* CXCR3^+^ Th1-like effector cells were decreased in third trimester vs. postpartum blood. The fact that these types of cells were largely absent in postpartum blood of relapsing MS patients implies their recruitment to the CNS to mediate disease activity. These findings were supported by a previous study, showing similar differences for *in vitro*-stimulated, IFN-γ-producing CD4^+^ but not CD8^+^ T cells (20). Because of the high levels of immunosuppressive hormones during pregnancy (24), it can be expected that a steep decline early after parturition (i.e., high postpartum relapse risk period) results in enhanced recruitment of Th1-like effector cells into the CNS of MS patients. In our HSNE analysis, we observed a similar but more moderate increase in *ex vivo* postpartum vs. third trimester effector memory Th frequencies in healthy controls. However, this was not found after taking more subtle proportional changes into account using manual gating. Despite the fact that parous females retain increased effector memory Th frequencies early after delivery (25), the larger nullipara count did not explain the lack of postpartum differences in the HC group.

Similar to pregnancy, discontinuation of disease-modifying regimen such as natalizumab often triggers severe MS relapses (26). This is likely due to the strong influx of brain-homing, pro-inflammatory immune subsets that accumulate in the peripheral blood of natalizumab-treated patients. Previously, we found that Th17.1 (IFN-γ^high^GM-CSF^high^IL-17^low^) cells are selectively enriched in the blood of MS patients who clinically responded to nalalizumab (19). In the current study, we found that the proportion of this subset was significantly lower in postpartum blood of relapsing MS patients, suggesting that Th17.1 cells have infiltrated the CNS to trigger a postpartum relapse. Similarly, Th17.1 frequencies were decreased in the blood of patients who rapidly develop clinically definite MS (19) and increased in MS brain tissues (19, 27). Since Th17.1 cells are highly refractory to hormones such as glucocorticoids (27), this subset could be the first to bounce back to their pro-inflammatory state after pregnancy to induce an MS relapse. Although freeze-thawing is known to have an effect on chemokine receptor expression (27), all samples used in this study were frozen and compared in a paired manner in our third trimester vs. postpartum analyses.

Additionally, we found that both the effector phenotype and CXCR3 expression of memory Th cells during pregnancy and in the early postpartum period are controlled by serum-related factors. These findings are supported by earlier studies showing that T-bet is downregulated *ex vivo* during pregnancy (28) and *in vitro* using high levels of pregnancy-induced progesterone and estradiol (29). We did not find differential levels of third trimester cortisol, estradiol and progesterone between patients and controls, which may explain that CXCR3 was downregulated on memory Th cells in both groups *ex vivo*. Since these levels did not correlate with CXCR3 expression or Th effector phenotypes in general, other pregnancy-relevant factors that were not investigated in this study, such as estriol (8) and early pregnancy factor (30) or differences in hormone sensitivity as discussed above could play a role as well.

Lastly, we found that memory Th cells of postpartum blood of relapsing patients produced increased levels of pro-inflammatory cytokines, which was already noticeable in the third trimester. This indicates that the proportion of pro-inflammatory Th cells that reside in the blood during pregnancy is higher in MS patients who will experience a relapse postpartum. Of all inflammatory cytokines analyzed, TNF-α, IFN-γ and GM-CSF production was the highest, but also differences in IL-17 were observed between clinical groups. This can be explained by the fact that CXCR3-expressing memory Th cells also include Th17 DP (IL-17^int^) and Th17.1 (IL-17^low^) cells (14).

Our study provides additional insights into how the suppression of CXCR3^+^ effector memory Th subsets during pregnancy corresponds to a postpartum relapse in MS. Due to the limited number of samples available for this study, our results should be validated in a larger cohort of similar well-defined treatment-naive pregnant MS patients, preferably with radiological data available to confirm the absence of immunological activity in cases not experiencing a clinical postpartum relapse. Since the composition of effector Th cells can easily be monitored in the blood, subsequent studies should clarify whether their pro-inflammatory nature could be evaluated as a prognostic cellular marker of disease activity during and after MS pregnancy.

## Data Availability Statement

The original contributions presented in the study are included in the article/Supplementary Material, further inquiries can be directed to the corresponding author/s.

## Ethics Statement

The studies involving human participants were reviewed and approved by Medical Ethics Committee Erasmus MC. The patients/participants provided their written informed consent to participate in this study.

## Author Contributions

SK performed experiments, analyzed data, interpreted results, and wrote the manuscript. RN organized the clinical study and critically revised the manuscript. AW-W, M-JM, AR, and RW performed experiments. CM assisted with the statistical analysis. WD, BB, and SB analyzed data and critically revised the manuscript. JS and ML designed the research, obtained funding, interpreted results, and critically revised the manuscript. All authors contributed to the article and approved the submitted version.

## Conflict of Interest

JS received lecture and/or consultancy fee from Biogen, Merck, Novartis, and Sanofi-Genzyme. The remaining authors declare that the research was conducted in the absence of any commercial or financial relationships that could be construed as a potential conflict of interest.
