# Supplementary material for: Effector T Helper Cells Are Selectively Controlled During Pregnancy and Related to a Postpartum Relapse in Multiple Sclerosis
Source: Front Immunol. 2021 Mar 15;12:642038. doi: 10.3389/fimmu.2021.642038 (PMC8005718; doi:10.3389/fimmu.2021.642038)
Supplement: Supplementary file 1 [file Table_1.pdf]

Supplementary table 1: Used FACS antibodies

**Marker**	**Clone**	**Fluorescent label**	**Company**
CCR4	L291H4	PE-Cy7	Biolegend
CCR6	G034E3	PE	Biolegend
CCR7	150503	PE-CF594	BD Biosciences
CD127	HIL-7R-M21	BV711	BD Biosciences
CD25	2A3	BV421 and BV605	BD Biosciences
CD3	SK7	AF700 and BV785	Biolegend
CD4	OKT4	BV510	Biolegend
CD38	HIT2	BV605	Biolegend
CD45RA	HI100	APC-H7	BD Biosciences
CD8	SK1	FITC, PerCP-Cy5.5	BD Biosciences
CXCR3	G025H7	APC	Biolegend
HLA-DR	G46-6	BB515	BD Biosciences
