# Supplementary material for: Effector T Helper Cells Are Selectively Controlled During Pregnancy and Related to a Postpartum Relapse in Multiple Sclerosis
Source: Front Immunol. 2021 Mar 15;12:642038. doi: 10.3389/fimmu.2021.642038 (PMC8005718; doi:10.3389/fimmu.2021.642038)
Supplement: Supplementary file 2 [file Image_1.pdf]

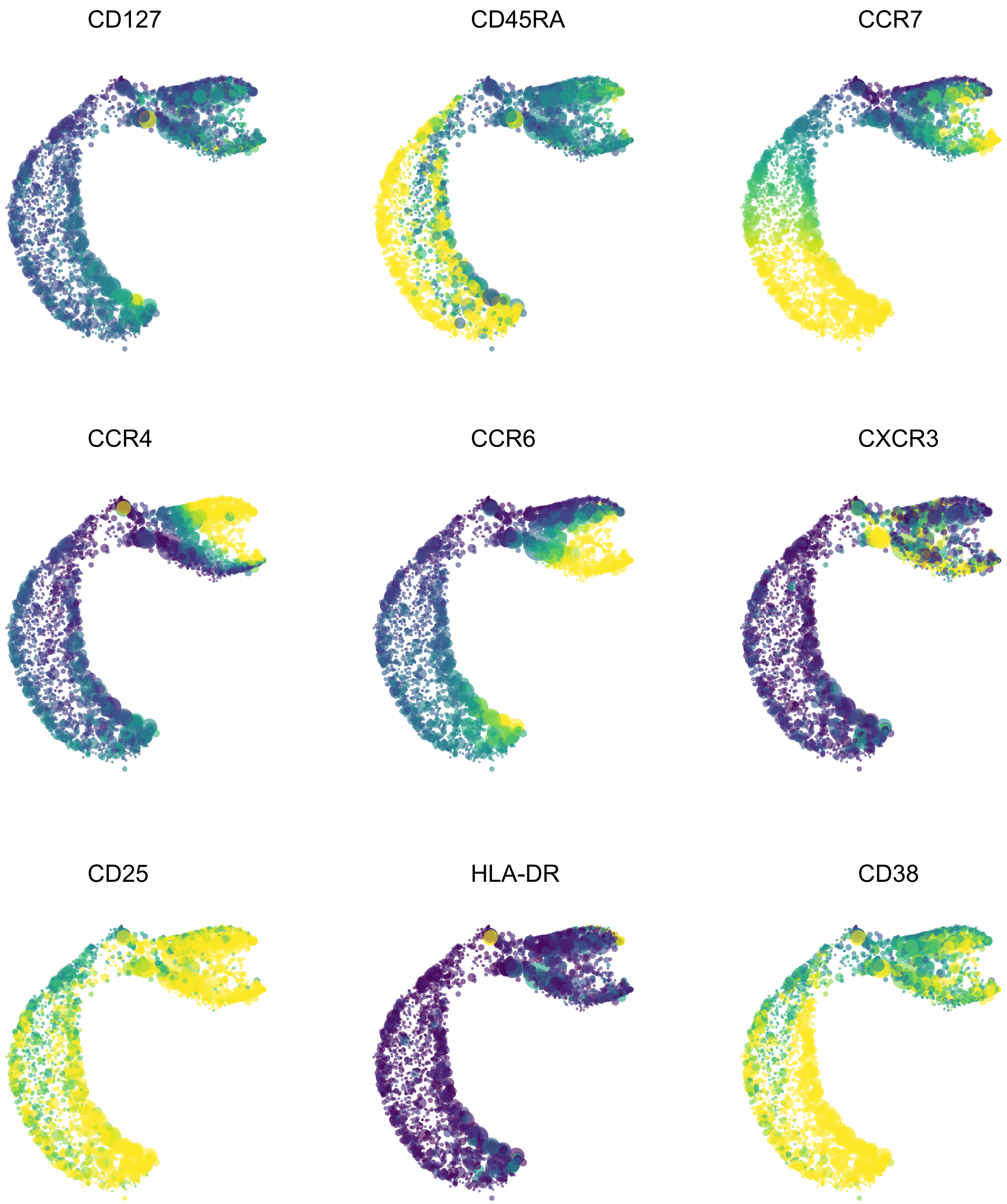

Supplementary Figure 1. Distribution of markers within the HSNE density plot of Th cells from non-relapsing patients and controls. HSNE plot of paired third trimester and postpartum CD4$^+$ Th cells of healthy controls (n = 12) and MS patients without a postpartum relapse (n = 13). CD127 etc. are shown within the cell clusters. Marker expression is based on the fluorescence intensity and displayed as a gradient (purple = low; yellow = high).
