# Supplementary material for: Effector T Helper Cells Are Selectively Controlled During Pregnancy and Related to a Postpartum Relapse in Multiple Sclerosis
Source: Front Immunol. 2021 Mar 15;12:642038. doi: 10.3389/fimmu.2021.642038 (PMC8005718; doi:10.3389/fimmu.2021.642038)
Supplement: Supplementary file 3 [file Image_2.pdf]

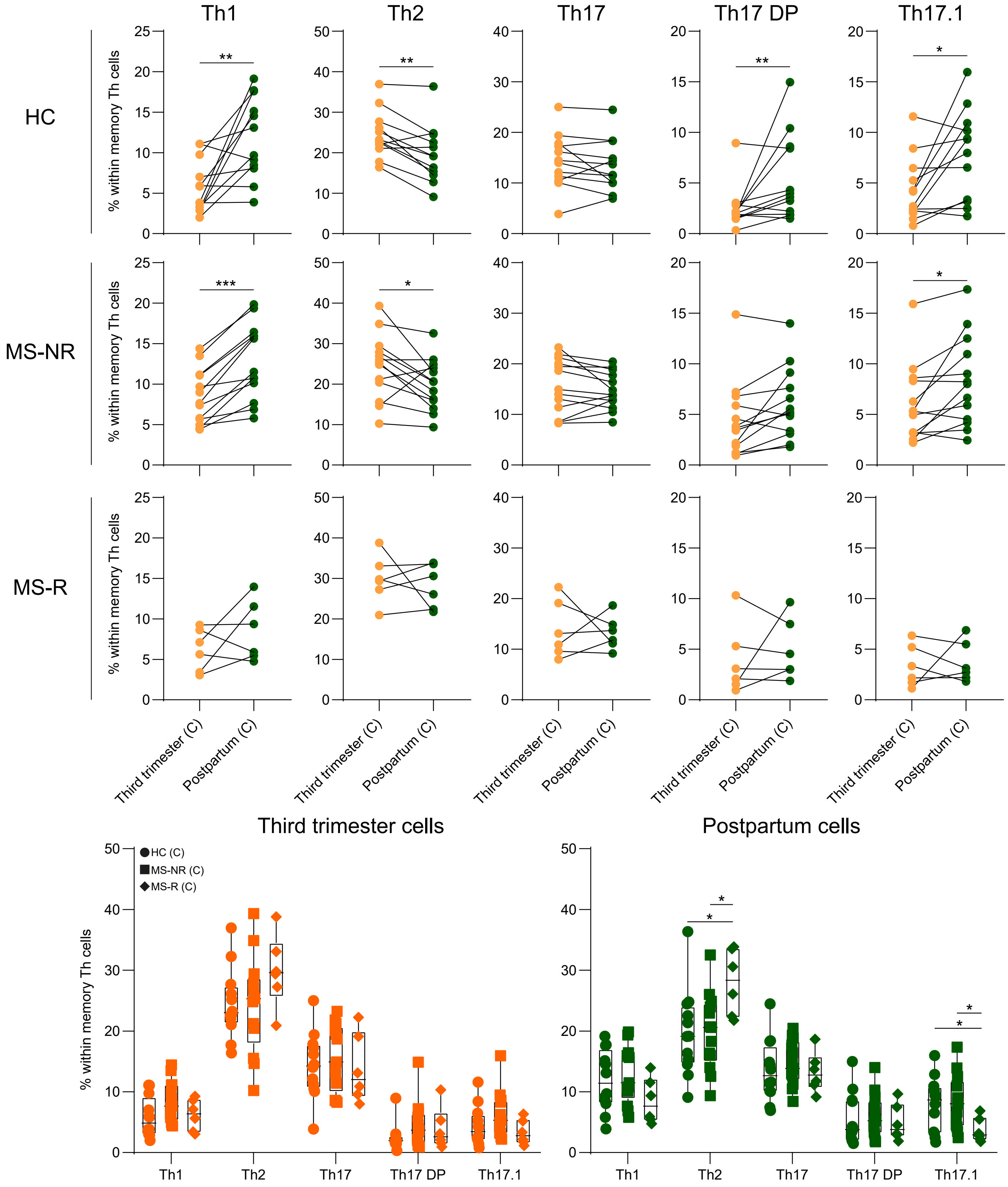

Supplementary Figure 2. Fluctuations of memory Th subsets in the third trimester and early postpartum period of patients and controls**.** Frequencies of Th1, Th2 Th17, Th17 DP and Th17.1 cells within memory Th cells were analyzed in paired third trimester and postpartum blood and each subset frequency was compared between clinical groups (HC, n = 12; MS-NR, n = 13 and MS-R, n = 6). Data were compared using GLMM with FDR-BH correction and 2-way ANOVA with Bonferroni's multiple comparisons tests *p < 0.05, **p < 0.01 and ***p < 0.001. 'HC' = healthy controls, 'MS-NR' = MS patients without a postpartum relapse, 'MS-R' = MS patients with a postpartum relapse and 'C' = cells.
