# Supplementary material for: Effector T Helper Cells Are Selectively Controlled During Pregnancy and Related to a Postpartum Relapse in Multiple Sclerosis
Source: Front Immunol. 2021 Mar 15;12:642038. doi: 10.3389/fimmu.2021.642038 (PMC8005718; doi:10.3389/fimmu.2021.642038)
Supplement: Supplementary file 4 [file Image_3.pdf]

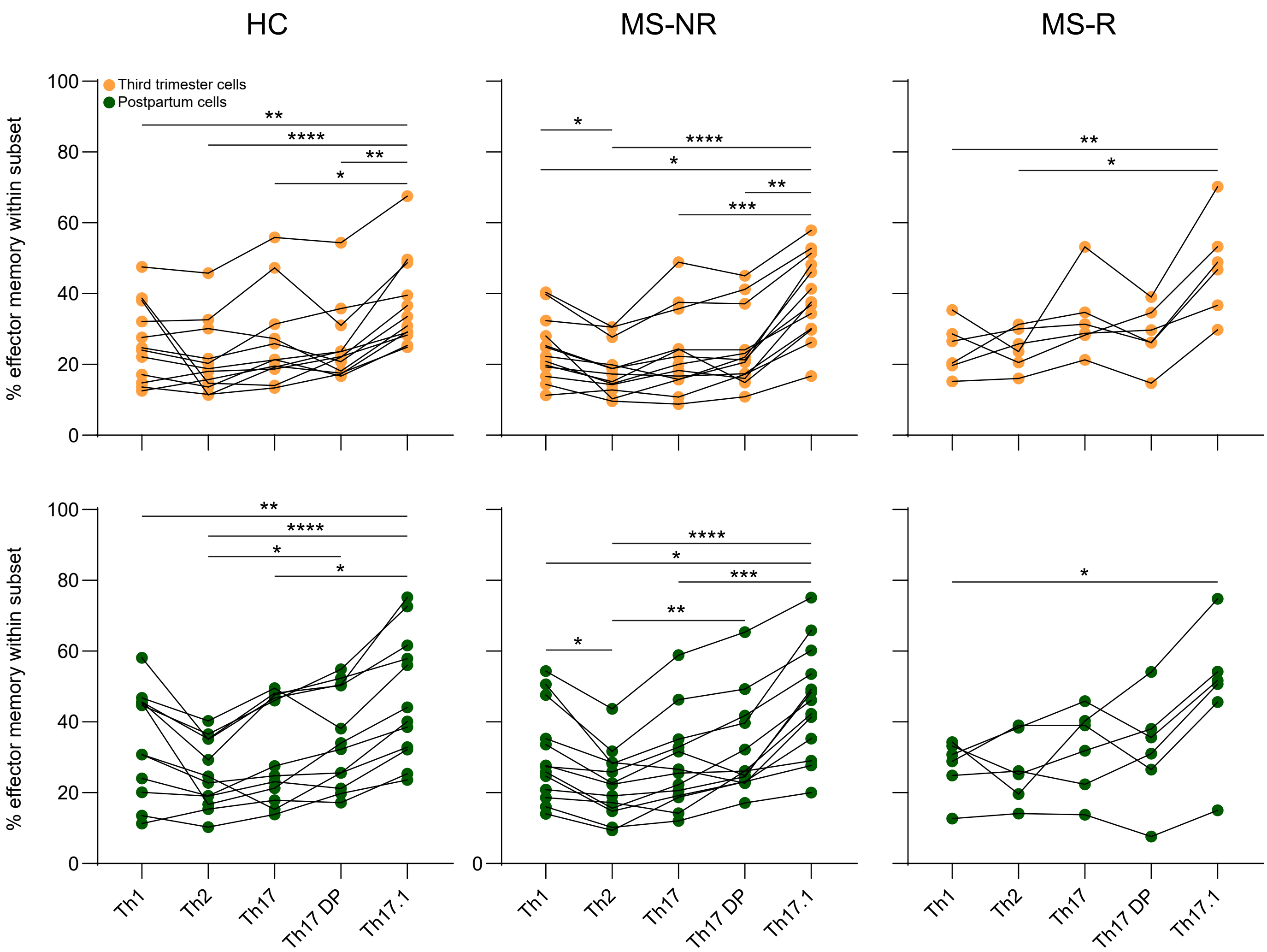

Supplementary Figure 3. Percentages within effector Th subsets from third trimester and early postpartum samples. Frequencies of effector memory cells within Th1, Th2, Th17, Th17 DP and Th17.1 subsets in third trimester and postpartum blood (HC, n = 12; MS-NR, n = 13 and MS-R, n = 6). Data were compared using Kruskal-Wallis with Dunn's multiple comparison tests *p < 0.05, **p < 0.01, ***p < 0.001 and ****p < 0.0001. 'HC' = healthy controls, 'MS-NR' = MS patients without a postpartum relapse, 'MS-R' = MS patients with a postpartum relapse.
