# Supplementary material for: Effector T Helper Cells Are Selectively Controlled During Pregnancy and Related to a Postpartum Relapse in Multiple Sclerosis
Source: Front Immunol. 2021 Mar 15;12:642038. doi: 10.3389/fimmu.2021.642038 (PMC8005718; doi:10.3389/fimmu.2021.642038)
Supplement: Supplementary file 5 [file Image_4.pdf]

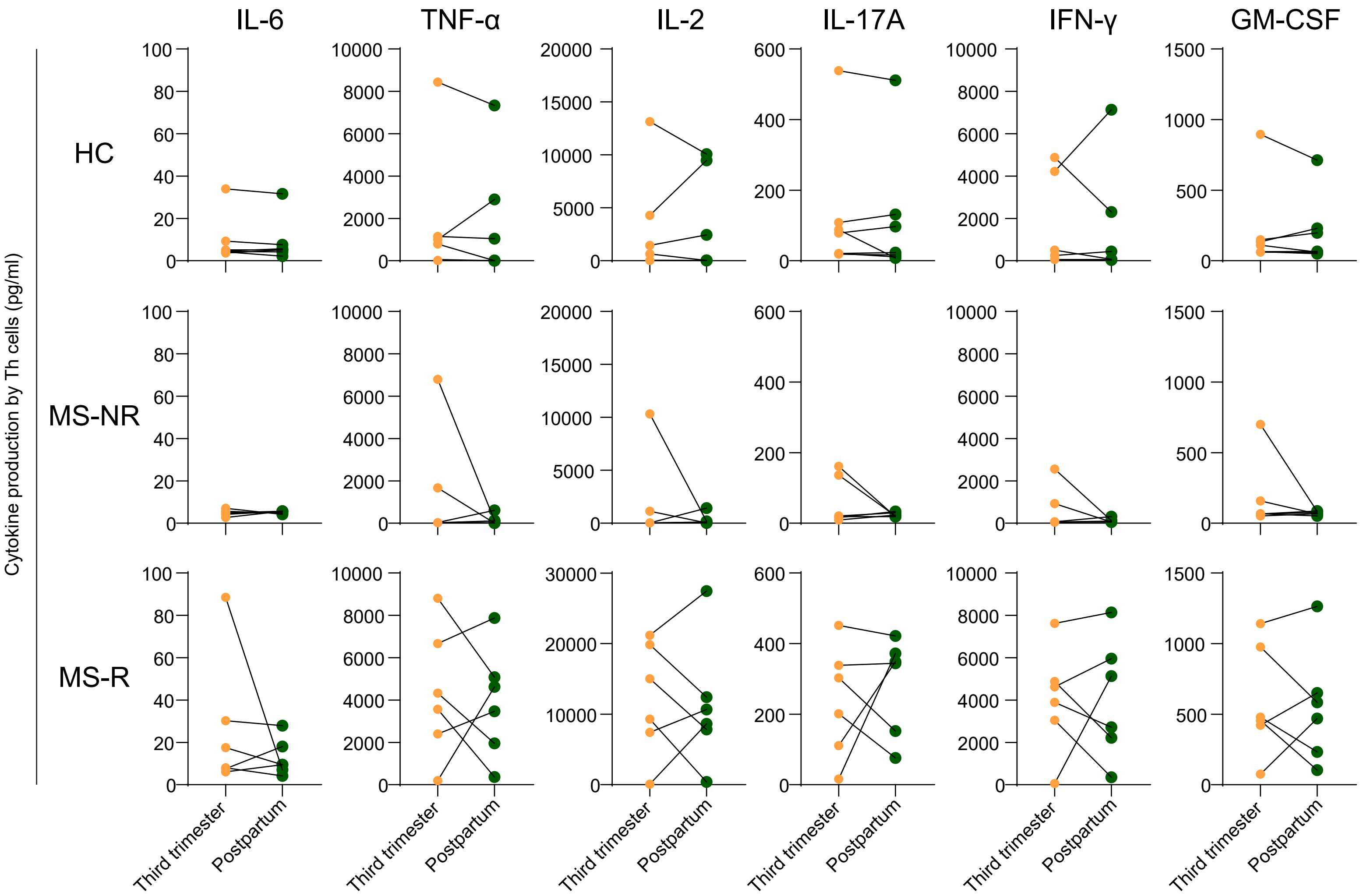

Supplementary Figure 4. Pro-inflammatory cytokine production by paired memory Th cells from third trimester and postpartum blood**.** IL-6, TNF-α, IL-2, IL-17A, IFN-γ and GM-CSF production (pg/ml) released by PMA/ionomycin-stimulated (5 hours) and FACS-sorted memory Th cells of paired third trimester and postpartum blood. Cytokines were measured in the culture supernatants and determined by Luminex (HC, n = 7; MS-NR, n = 6 and MS-R, n = 6). Data were compared using GLMM with FDR-BH correction. 'HC' = healthy controls, 'MS-NR' = MS patients without a postpartum relapse, 'MS-R' = MS patients with a postpartum relapse.
